# Diagnostic Potential of FT-IR Fingerprinting in Botanical Origin Evaluation of *Laurus nobilis* L. Essential Oil is Supported by GC-FID-MS Data

**DOI:** 10.3390/molecules25030583

**Published:** 2020-01-29

**Authors:** Stella A. Ordoudi, Maria Papapostolou, Stella Kokkini, Maria Z. Tsimidou

**Affiliations:** 1 Laboratory of Food Chemistry and Technology (LFCT), School of Chemistry, Aristotle University of Thessaloniki (AUTh), 54124 Thessaloniki, Greece; steord@chem.auth.gr (S.A.O.); papaposm@chem.auth.gr (M.P.); 2 Laboratory of Systematic Botany and Phytogeography (LSBPh), School of Biology, Aristotle University of Thessaloniki (AUTh), 54124 Thessaloniki, Greece; kokkini@bio.auth.gr

**Keywords:** *Laurus nobilis* L., bay laurel, essential oil, 1,8-cineole, FT-IR spectroscopy, GC-FID-MS, green analytical methods, chemometrics, fingerprinting, botanical origin

## Abstract

The last years, non-targeted fingerprinting by Fourier-transform infrared (FT-IR) spectroscopy has gained popularity as an alternative to classical gas chromatography (GC)-based methods because it may allow fast, green, non-destructive and cost-effective assessment of quality of essential oils (EOs) from single plant species. As the relevant studies for *Laurus nobilis* L. (bay laurel) EO are limited, the present one aimed at exploring the diagnostic potential of FT-IR fingerprinting for the identification of its botanical integrity. A reference spectroscopic dataset of 97 bay laurel EOs containing meaningful information about the intra-species variation was developed via principal component analysis (PCA). This dataset was used to train a one-class model via soft independent modelling class analogy (SIMCA). The model was challenged against commercial bay laurel and non-bay laurel EOs of non-traceable production history. Overall, the diagnostic importance of spectral bands at 3060, 1380–1360, 1150 and 1138 cm^−1^ was assessed using GC-FID-MS data. The findings support the introduction of FT-IR as a green analytical technique in the quality control of these often mislabeled and/or adulterated precious products. Continuous evaluation of the model performance against newly acquired authentic EOs from all producing regions is needed to ensure validity over time.

## 1. Introduction

Essential oils (EOs) are natural products with multifunctional properties of great interest for the pharmaceutical, cosmeceutical and food industry. For example, food-grade EOs with spicy and herbal flavors that can impart the essence of the plant source from which they derive are also highly appreciated in meat, sauces, bakery and beverage manufacture for antimicrobial and antioxidant activities [1]. Among them, the bay laurel or sweet bay EO is of industrial demand not only because of its aromatic, spicy flavor but also for its recognized antioxidant-antimicrobial activities [2]. The plant source of this EO is the dry leaves of the species *Laurus nobils* L., a seasoning that is well-known to consumers all over the Mediterranean and East Asia region where the tree is natively grown and extensively cultivated (Turkey, Morocco, Israel). Commercial products with similar common names such as California bay (*Umbellularia californica*), mountain laurel (*Kalmia latifolia*), bay rum tree or West Indian bay tree (*Pimenta racemosa*), Mexican bay (*Litsea glaucescens*), Indian bay (*Cinnamomum tamala*) and Indonesia bay leaf (*Syzygium polyanthum*) are of totally unrelated botanical origin and can even pose health risks if used as food seasonings [3].

Evaluation of the botanical origin of the EOs is of fundamental importance in their integrity studies [4]. It is a challenging area of research because the chemical profile of these products can be characteristic of specific plant genotype but also vary due to several biotic and abiotic factors (e.g., the stage of plant development, the part of the plant) as well as post-harvest treatments and extraction means [5]. Regarding *L. nobilis* EO, the effect of plant organ [6,7], gender [8], phenological stage [9], post-harvest handling [10] and EO production methods [11,12] have been examined to a certain extent.

Analysis of the chemical composition of EOs is carried out using gas chromatography (GC) coupled with flame ionization detectors (FID) or mass spectrometers (MS). According the GC data published so far, *L. nobilis* leaf EO is dominated by 1,8-cineole that usually ranges from 11% to 64% *v*/*v*. It also contains α-terpinyl acetate (traces—27%), linalool (traces—18.5%), methyleugenol (traces—19%), sabinene (0.3%—12%), α- and β-pinene (traces—7.5%, each), terpinen-4-ol (traces—5%) and many other minor constituents and artefacts [13].

Fourier-transform mid infrared (FT-MIR) spectroscopy coupled with chemometrics is appraised as a non-destructive and versatile means for the evaluation of food integrity [14]. Regarding EOs, FT-IR fingerprinting has gained increasing popularity as an alternative, green, fast, and cost-effective approach for the evaluation of different quality aspects, e.g., grading according to their content in major volatile constituents [15,16], toxic compounds [17] or suspected fragrance allergens [18], but also detection of geographical origin [19] and identification of counterfeit [20]. As far as it concerns applications of bay laurel EO studies, the literature is extremely limited [20,21,22].

The objective of the present study was to explore the diagnostic potential of FT-IR fingerprinting of *L. nobilis* EO for the identification of its botanical integrity. A one-class classification strategy was adopted. For such an aim, a reference FT-IR spectroscopic dataset using EOs obtained from taxonomically identified bay laurel leaves was necessary. Commercial bay laurel and non-bay laurel EOs with varying content in 1,8-cineole (eucalyptus, rosemary, sage, melissa) were also included in the study. An exploratory analysis of the spectral data was accomplished using principal component analysis (PCA) and soft independent modeling of class analogy (SIMCA). Where necessary, GC-FID-MS analyses were also carried out to offer compositional information that supported the FT-IR findings.

## 2. Results and Discussion

### 2.1. Assignment of FT-IR Transmittance Spectra

Spectra of the reference bay laurel leaf EOs in the mid-infrared region (4000–400 cm^−1^) presented characteristic fringing patterns especially in the regions above 3000 cm^−1^, between 2800 and 1750 cm^−1^ and also in the low-resolution region between 1600 and 900 cm^−1^. Second order derivatization of the spectra allowed to corroborate chemical assignment of overlapped spectral bands of zero order ones (Figure S1).

**Figure 1:**
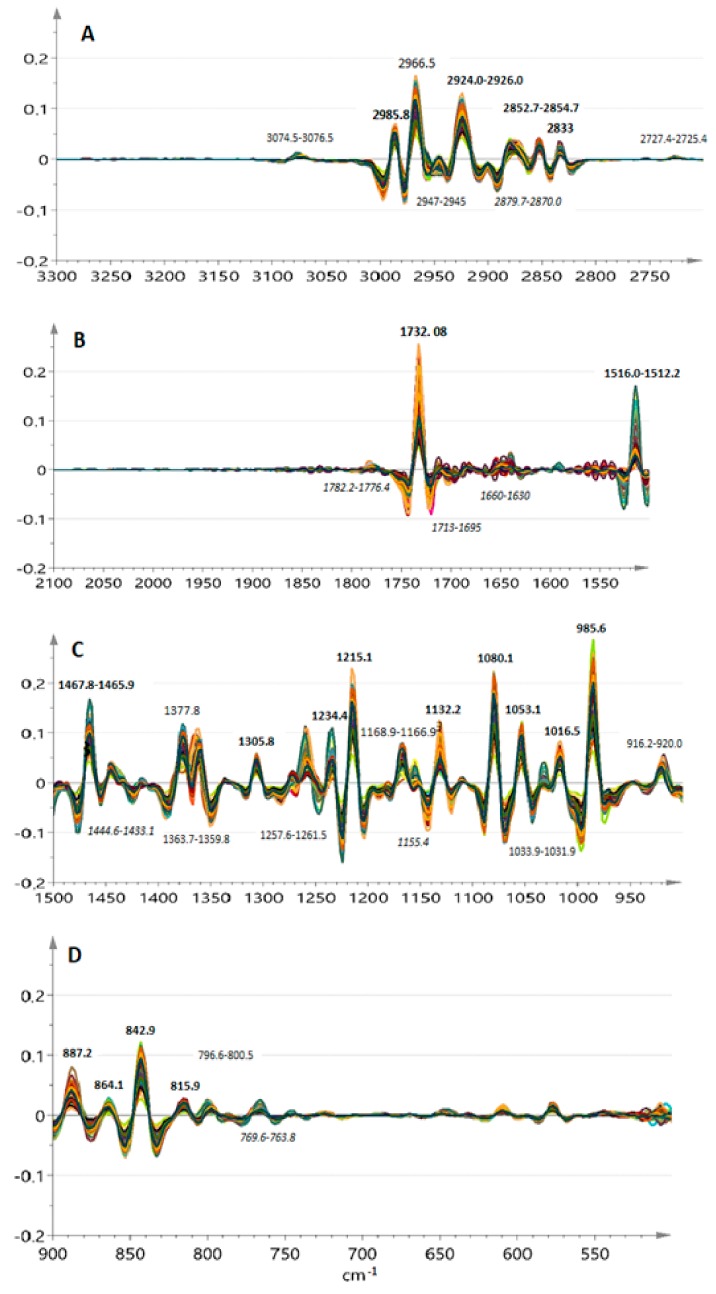
Inverse 2nd order derivative FT-IR transmittance spectra of the reference bay laurel leaf essential oil (EO) samples (*n* = 97); 3300–2700 cm^−1^ (**A**); 2100–1500 cm^−1^ (**B**); 1500–900 cm^−1^ (**C**); 900–500 cm^−1^ (**D**). Peaks shifted by more than ± 2 cm^−1^ are shown in italics.

Figure 1A–D displays the overlaid second derivative spectra of the 97 reference EO samples in several sub-regions within the range 3300–600 cm^−1^. Highly noisy parts of the spectra or regions devoid of signals were excluded to ease visual inspection of the spectral features. Small regions where shifts in the frequency of bands surpass the instrumental resolution (4 cm^−1^) are pinpointed to show a higher degree of variance among the samples. Table 1 summarizes these data and provides information about peak assignments based on the comparison with corresponding spectra of reference compounds and the literature data.

The overlaid derivative spectra were almost identical especially in the region 600–1500 cm^−1^. This region describes better the skeletal vibrations of the EO constituents and, therefore, the chemical fingerprint of the reference samples. Inspection of the band shape and position within narrower regions associated with the carbonyl and double bond (1630–1780 cm^−1^) as well as the methyl/methylene group vibrations (e.g., 2800–3100, 1430–1445 and 1360–1380 cm^−1^) indicated different patterns in variance among samples. The relative intensities of the bands also varied a lot (Figure 1) highlighting differences in the relative contents of the corresponding structural features that could not be further interpreted by visual examination.

### 2.2. Spectral Data Pretreatment and Variable Selection

It is well accepted that application of different pretreatment methods to the raw spectroscopic data has a large effect on the diagnostic efficiency of the chemometric model [26]. The performance of each pretreatment is usually evaluated in the course of pre-trials and it is related to the type of multivariate analysis that will be carried out next. In our study, the smoothed and derivatized FT-IR spectra were further pre-treated with the Pareto scaling method, which uses the square root of the standard deviation as the scaling factor ($x_{\mathrm{ik}}-{\bar{x}}_{k}$/$\sqrt{s_{k}}$) to reduce the importance of large values due to the most abundant vibrations [26].

The pretreated dataset contained 1816 variables (the whole spectrum) for each of the 97 bay laurel EO samples (1816 × 97). Its dimensionality was considerably reduced after principal component analysis so that the first five principle components (PCs) to account for the 84.1% of the variance in the original data. However, the Hotellings’ *T*^2^ test indicated the presence of one clear outlier, which caused data overfitting. The variable contribution plots showed that this sample outperformed mainly because of outranging intensity of its spectral bands at 887, 972, 1547–1572, 1640–1718, 2854 and 2926 but also at 3075 cm^−1^ indicating possibly extended the conjugation of the carbonyl group with aromatic ring systems or different vibration patterns of methyl groups. After its exclusion, a new outlier revealed the outranging spectral bands in the region 2200–2400 cm^−1^ which were possibly due to dissolved carbon dioxide. This sample was not excluded from the dataset, but the specific region was excluded from subsequent analysis. The latter was carried out using interval-PCA (i-PCA) after skipping also other highly noisy or shifted signals (500–600, 1520–1700, 1760–2800 and 3100–4000 cm^−1^) (Figure 1).

The data in the remaining spectral bands (*n* = 662 variables) were then grouped in 20 intervals each of which was independently analyzed for possible similarities and differences through PCA using 3-PC models. Percentage of explained variance helped to evaluate the performance of each interval. The PC scatterplots assisted visualization of those sample sub-groupings, which were promising for the aim of the study (Figure S2). As a result, six distinct sub-regions (600–658, 1138–1414, 1713–1760, 2800–2868, 2930–2986 and 3045–3100 cm^−1^) were selected as the most informative ones. In retrospect, it was observed in Table 1 that this reduced dimensionally dataset (*n* = 296 variables) included the most characteristic vibrations of bonds of the major EO constituents. In this dataset, bay laurel EO constituents that bear benzene rings (e.g., methyleugenol, eugenol or isomers of cymene) are expected to be represented by vibrations of their alkyl or ether group substituents and not of their ring double bonds (1640–1660 cm^−1^). Isolated or cyclic double bonds (e.g., in cyclohexene rings) are not represented. Information about carboxylic ester bonds such as those in α-terpinyl acetate or even alkyl ketone groups is retained. The variable selection procedure helped to filter out frequencies of sp^2^ C–H bending vibrations (652–1136 cm^−1^) and retain stretching ones that indicate more specifically the effect of adjacent oxygen or other electronegative groups (see Table 1). Subtle differences in the spectral interval between 600 and 660 cm^−1^ were also highlighted as informative although their chemical assignment is tricky; they could represent vibrations of alkyl–halogen or alkyl–sulphur bonds or generated by deformation of the –COO^−^ group in acetate esters [23,24,25]. This dataset (296 variables, 96 samples) was then used for further analysis.

### 2.3. Exploratory Analysis of Spectral Data

**Figure 2:**
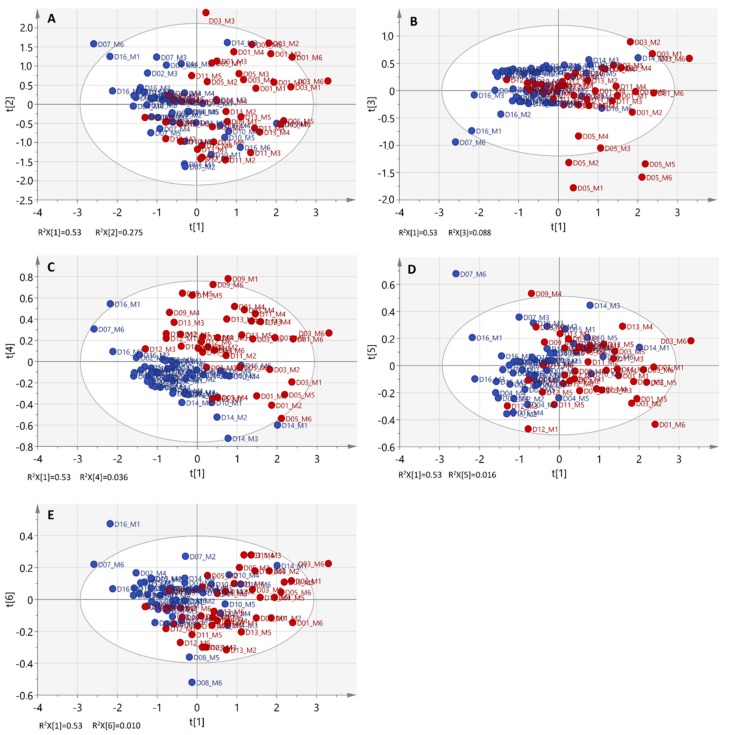
Scatterplots of principle component analysis (PCA) scores (t) along the first and second (**A**), third (**B**), fourth (**C**), fifth (**D**) or sixth (**E**) principal components extracted from the pre-processed dataset of 88 reference bay laurel EOs (obtained from leaves of cultivated trees in AUTh campus). •: female (*n* = 8), •: male trees (*n* = 7). Dot codes refer to the tree (D#) and harvest month (M#) identifiers.

PCA performance was clearly improved after variable selection (five PCs accounted for the 94.1% of the total variance) and no outliers were revealed. When projected on the scoreplot of the first two PCs, the reference samples formed a cloud of points with no clear sub-groupings among them. This was interesting because the included EOs have been obtained from leaves harvested within a period of six months (April–September) from both female and male trees (see 3.1) (Figure 2A–Ε).

**Figure 3:**
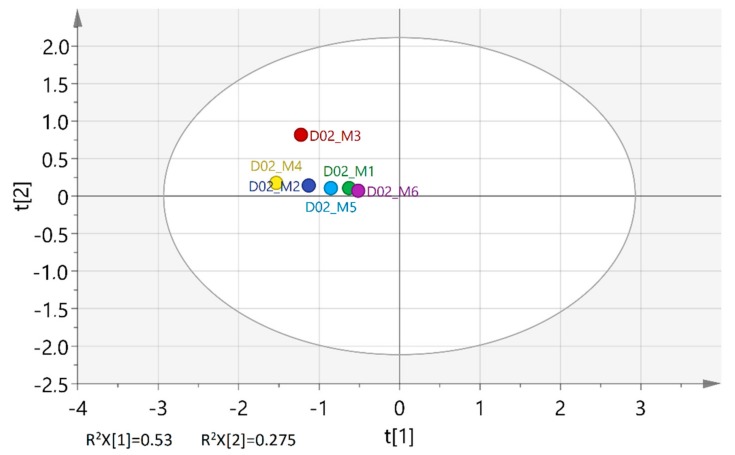
Scatterplot of PCA scores (t) along the first and second principal component of bay laurel leaf EO samples corresponding to six different sampling dates (M1–M6) from tree D02 (AUTh campus).

Inspection of the PCA scoreplots in Figure 2A did not show any patterns of discrimination according to the sampling date of the plant material that might correspond, among other factors, to different phenological stages of the 15 trees. For better visualization, Figure 3 displays an example of how the EOs from leaves of a given tree source were scattered along the first two PCs throughout the leaf sampling period. From a diagnostic point of view, the sample that differed from the rest due to its higher PC2 score value owed its variance to spectral vibrations near 1150 cm^−1^ although there may be several other spectral features that contribute to the formation of this PC (e.g., the bands at 1377, 1368, 1356 cm^−1^). The particular EO sample refers to leaves harvested in June (M3), after flowering and at the onset of fruit development on the tree. To gain better insight to the FT-IR spectroscopic findings, the GC-MS chromatographic profiles of these particular EOs were also obtained (Table 2, Figure S3).

The mass spectral data of the chromatographic peaks helped to identify the chemical constituents of those samples and estimate the relative contents (Table 2). Thus, a total number of 27 to 63 compounds were identified in EOs produced throughout this period. Despite seasonal variation, percent areas of peaks corresponding to 1,8-cineole and α-terpinyl acetate had always the same order of magnitude and represented the major constituents of the samples (45%–60% of the total peak area), as expected. Noticeably, the sample with distinct FT-IR spectral features at around 1150 cm^−1^ was poorer in 1,8-cineole and α-terpinyl acetate (47% in total) but had the richest profile in less abundant volatile constituents like sabinene, bornyl acetate and several others. GC-MS data provide evidence for seasonal changes in the chemical composition of these EO samples that account partially for variance in the FT-IR spectral data explained mainly by the two first PCs.

**Figure 4:**
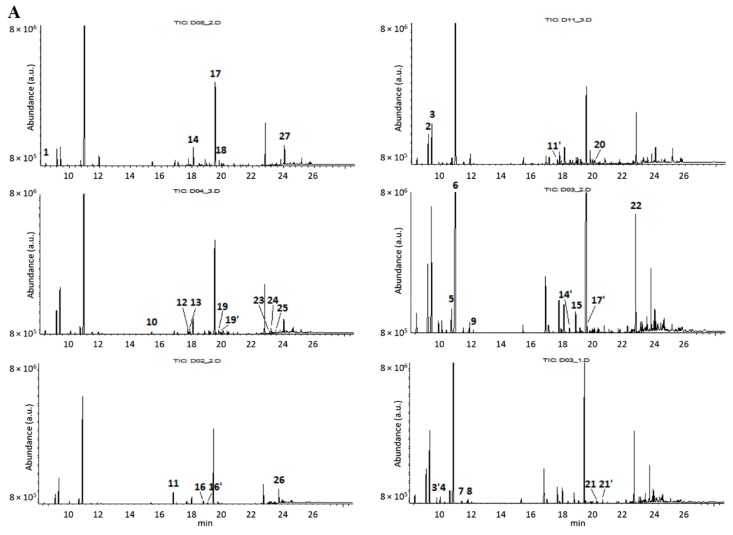

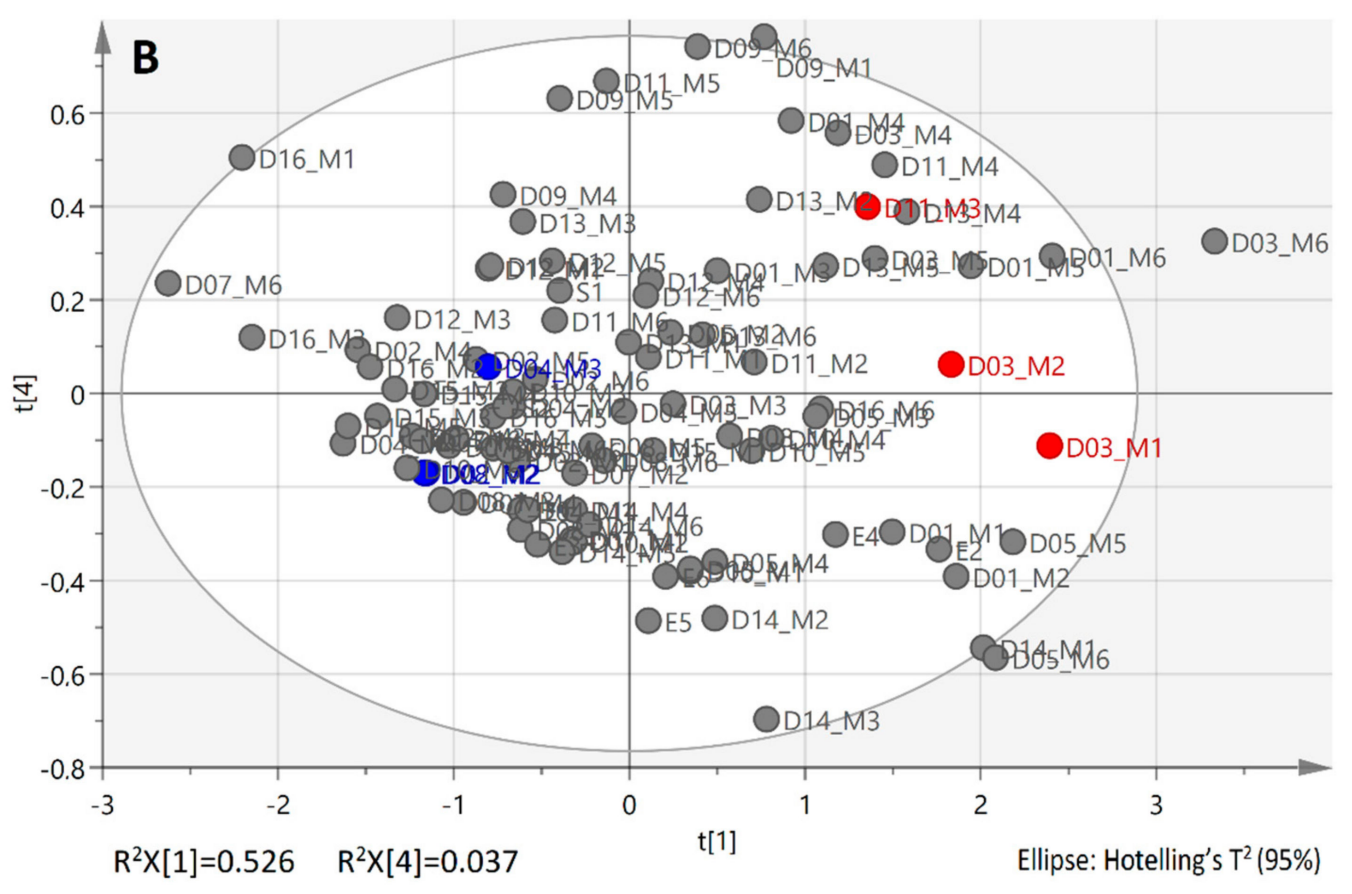
GC-MS chromatographic profiles of randomly selected EOs originated from three female and three male bay laurel leaves (**A**); projection of the same EO samples on the PC14 scoreplot of the reference FT-IR dataset (**B**). •: female trees •: male trees. Dot codes refer to the tree (D#) and harvest month (M#) identifiers. The chromatographic peaks were cross-referenced against the NIST mass spectral library (version 2.0f, 2008) and assigned to compounds no 1–27, as shown in Table S1.

Other PCs that do not explain the high percentage of variance among the data but might hide meaningful information [27] were also explored in this study to investigate patterns of intra-species variability of *L. nobilis* L. (Figure 2B–E). Indeed, the fourth PC (R^2^(X) = 3.56%) was found to be more likely to contain information relating to the gender of the tree (Figure 2C). More than 90% of the samples from female trees were projected close to each other and formed a distinct group of points with negative PC4 score values. On the other hand, 75% of their male counterparts were spread across the plot with positive PC4 score values. The recognized pattern of distribution was most probably associated with variance in the spectral band intensity and location around 3060, 1150 and 1138 cm^−1^. The potentially diagnostic importance of these bands was verified after examination of bay laurel EOs from leaves of two wild-grown female trees from a mountainous site (Athamanon mountain, Epirus GR, 1150 m). These two samples were found to be distributed along PC4 close to the other female AUTh-originating ones (t4 = 0.221, −0.025). Their grouping in the hyperspace of the first, second and fourth PCs (plots available, not shown) was similar. This result implied that the explained variance by the fourth PC is relevant to gender-specific differences among the leaf sources of the reference set despite geographical-climatic effects. GC-MS profiling of several EOs (Figure 4A) that were greatly scattered on the PC14 scoreplot (Figure 4B) was then carried out to investigate further the observations.

The selected EOs mostly derived from leaves that had been collected during the May–June season. The GC data of EOs (Table S1) from male trees regarding relative contents in 1,8-cineole plus α-terpinyl acetate differed some way but the size of difference was affected mainly by seasonal changes, possibly because gender-related differences are not expected to be expressed in leaves to the same extent as in other dedicated organs (flowers) [28]. Such a hypothesis needs further examination. Overall, GC-MS data highlighted that the relative contents of highly abundant constituents like α-terpinyl acetate, methyleugenol and linalool lead to variance of diagnostically important FT-IR bands at 3060, 1150 and 1138 cm^−1^.

It is worth mentioning that EOs from commercial leaf products (E1–E6) for which botanical species were certified in this study were also included in the reference FT-IR dataset, though no information about their gender or harvest season was known. These samples were grouped together with the rest of reference samples that were used to model the class of “authentic bay laurel EOs” (Figure S4).

### 2.4. One-Class Classification

Defining the boundaries around a target class such as the “authentic bay laurel EOs” is a major challenge of the one-class classification strategy. A successful model will be able to recognize as many true-positive inputs as possible while minimizing false-positive ones [29]. In the present study, the reference FT-IR dataset was used to train a one-class SIMCA model and evaluate it against EOs of non-traceable production history. Analysis was based on measuring whether their residual variance exceeds the boundaries of the “authentic bay laurel EOs” class, as explained below.

**Figure 5:**
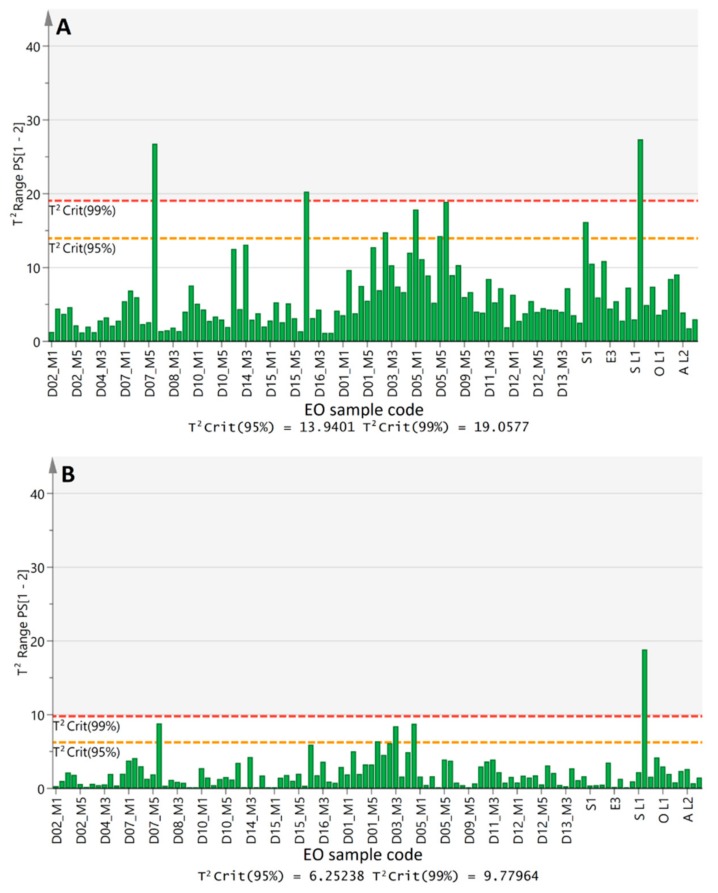
Hotelling’s *T^2^* plots displaying the predicted variance of commercial bay laurel EOs from the one-class model of reference EOs explained by six PCs (**A**) and two PCs (**B**)**.**

EO products labeled either as *L. nobilis* L. or as “Bay laurel” and “Daphne” were analyzed first (*n* = 11) (Table S2). The spectral data were acquired and pre-processed as previously reported. Figure 5A displays the measured variance from the PCA modelled class (6 PCs, R^2^(X) = 95.4%) of authentic bay laurel EOs in terms of the Hotelling’s *T*^2^ test. One out of the 11 commercial samples was found to exceed the critical *T*^2^ value (*p* < 0.01) that forms the upper boundary of the class. Closer inspection of the results showed that its distance from the hyperspace of the 6-PC model was equal to that of two reference samples (D07 M6, D16 M1) in Figure 5A. Given that the PCA model retains meaningful information about intra-species variation in higher order PCs (e.g., third, fourth PC), we considered these axes of the hyperspace as less important for one-class classification. Consequently, only the first two PCs, which explain the highest percentage of data variance among all reference bay laurel EOs (R^2^(X) = 79.7%), were investigated for fitness for purpose. Indeed, *T*^2^ range values of the one-class model lowered (9.8 vs. 19.1, *p* < 0.01) after the exclusion of the lower axes (Figure 5B). Only one non-compliant sample was detected (VL1, Figure 5B).

The particular EO had been purchased directly from a manufacturer, who provided certificate of GC-FID analysis from an accredited laboratory. GC-FID-MS analysis of this sample in the present study verified, under similar elution conditions, that it was richer in individual constituents, especially 1,8-cineole and α-terpinyl acetate (60%). The relative contents in other major volatile constituents such as sabinene as well as minor ones, e.g., γ-terpinene, β-pinene, *p*-cymene, (+)-3-carene,(+)-2-carene signified higher content in monoterpene hydrocarbons (Figure S5), a finding possibly related to the fact that shoots and not only leaves were extracted. Inspection of its FT-IR spectral characteristics and analysis of the distance to the model contribution showed that a series of spectral bands within the fingerprint region of the IR spectrum were outranging, most probably due to the abundance of the corresponding structural moieties (data available, not shown). Noteworthy, another sample (VL2) that was provided by the same manufacturer few months later—with the confirmation that it belonged to the same batch—did not deviate as the previous one and it was grouped together with the authentic bay laurel EOs (Figure 5). The results from this evaluation test designated that except for extraction means, other parameters may affect correct classification.

**Figure 6:**
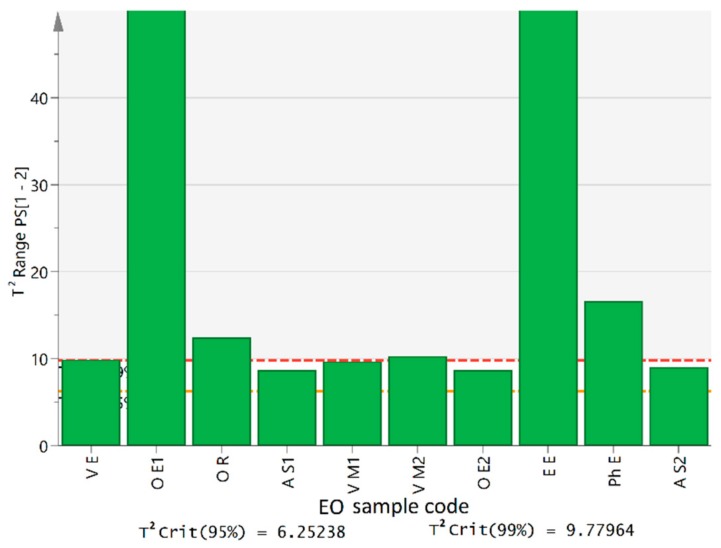
Hotelling’s *T^2^* plot displaying the predicted variance of all the commercial EOs from the one-class model of reference bay laurel EOs (S L1—V L2); eucalyptus EOs (V E—Ph E), rosemary (O R), sage (A S1, A S2) and melissa EOs (V M1, V M2) EOs.

A series of non-bay laurel leaf EOs labeled as eucalyptus (*n* = 5), rich in 1,8-cineole (68%–92% *v*/*v* according to GC-FID data) were also examined by the model. None of the samples was classified as bay laurel EO at the 95% confidence level (Figure 6) verifying the sensitivity of the model, which does not only depend on the content of major compounds but also on the whole fingerprint of each EO (Figure S6). Further verification of the model sensitivity was sought using three other types of commercial EOs that contained 1,8-cineole from practically zero (melissa EO) to around 45% (*v*/*v*) (rosemary, sage EOs). Apart from this major compound, these EOs differed also in the overall GC-MS chromatographic profile (Figure S6), in accordance to literature [30,31]. Differences in chemical composition were clearly illustrated in their FT-IR spectra, over the regions that were evaluated in this study (Figure S7).

## 3. Materials and Methods

### 3.1. EOs from Collected and Taxonomically Identified L. Nobilis Leaves (Reference Bay Laurel EOs)

Ninety seven samples that were regarded as reference had been all produced in the LSBPh with the classical 3 h hydrodistillation method described in the European Pharmacopoeia [32] using a Clevenger-type apparatus. The obtained EOs were collected in glass vials and stored at 4 °C, protected from light and oxygen until their analyses.

Only dry leaves, after collection from plants cultivated as ornaments (*n* = 91) or purchase from the market (*n* = 6), were used as starting material. The former had been harvested randomly from tag trees or shrubs that are spread across a restricted urban area of ca. 0.2 hectares (garden of AUTh campus), (*n* = 15) or at a mountainous site (Athamanon mountain, Epirus, Greece) (*n* = 2). Sampling was carried out at six dates from early April (after flowering) to early September (fruit ripening) from trees that differed in gender and phenological stage of growth. Τhe freshly picked bay laurel leaves were air-dried in shade at ambient temperature for a minimum period of one month before the isolation of EOs [33]. Voucher specimens from the collected plants were deposited in the Herbarium of Biology School at Aristotle University of Thessaloniki.

The commercial dry leaves were purchased from the market of Thessaloniki and were taxonomically identified to belong to the *L. nobilis* species. Apart from labeled information about the country of origin (Greece) and suggestions about their possible collection site (personal communication with retailers), no other tracking data about the tree source were available.

### 3.2. Commercial EOs

Commercial EO products were purchased from Thessaloniki market (herbal shops, producers, pharmacies, Thessaloniki, Greece) (*n* = 21). All products already packed in amber glass vials were stored at 4 °C until analysis. Details on labeling information are shown in the Supplementary Materials (Table S1).

### 3.3. Solvents and Standards

GC-grade dichloromethane (≥99.9%) was purchased from Honeywell (Charlotte, NC, USA), *n*-hexane (analytical grade, ≥99%) and acetone (HPLC grade, 99.8%) were from Chem-lab (Zedelgem, Belgium). The standard 1,8-cineole (food-grade, ≥99%), methyleugenol (food-grade, ≥98%) and α-pinene (98%) were from Sigma-Aldrich (Sigma-Aldrich Chemie GmbH, Steinheim, Germany).

### 3.4. FT-IR Transmission Spectroscopy

FT-IR spectra were acquired using a Shimadzu IRAffinity-1 (Shimadzu Europa GmbH, Duisburg, Germany) spectrometer operating in the region 4000–500 cm^−1^ in transmittance mode and located in an air-conditioned room (25 °C) (LFCT). An aliquot (10 μL) of each EO sample was placed between two rectangular ZnSe windows (41 mm × 23 mm, 2 mm thick) with the aid of a 25 μL GC syringe (Hamilton, NV, USA). The pair of windows was then fixed without the use of PTFE spacers in a demountable liquid transmission cell (Omni-Cell^®^, Specac Ltd., Orpington, UK), according to manufacturer’s instructions. A total of 64 scans at 4 cm^−1^ resolution were acquired for each spectrum. Each sample was scanned in triplicate against a background air spectrum. Before each measurement, ZnSe windows were thoroughly cleaned with successive use of hexane and acetone and wiped out with clean and soft tissue. Spectra were stored and pre-processed using the software IRsolution (version 1.50) supplied by the same manufacturer.

### 3.5. FT-IR Spectral Data Analysis

#### 3.5.1. Data Preprocessing

All the raw spectra were first smoothed by 10 points using the software function ‘‘smoothing action’’. Then the ‘‘derivative action’’ facility was selected to calculate second-order derivatives using the Savitzky–Golay method and 11 data points as the interval. Re-scaling of the spectra by inversion and multiplication were achieved using the ‘‘arithmetic action’’ and multiplying each data point by a factor of (−100). The data were extracted, combined and further processed with Microsoft Excel 2013 software (Microsof Corp., Redmond, WA, USA) to produce a single dataset that comprised of 1816 columns with numerical data per row (average instrumental response at each wavenumber). Each row represented a different EO sample. The dataset of taxonomically identified bay laurel leaf EOs (*n* = 97) was regarded as the reference.

#### 3.5.2. Selection of Variables

The regions devoid of signals were skipped first. The data in the remaining regions were then processed with the aid of i-Toolbox of MatLab2011 7.12.0 (The MathWorks Inc., Natick, MA, USA). In particular, interval-Principal Component Analysis (i-PCA) was performed with data from 20 intervals of continuous wavenumbers and constant width (*n* = 30). In this case, the classical methodology of PCA that transforms a set of variables into a new set of composite variables, the principle components (PCs) using linear combinations of the original variables, was extended to the interval data [34]. The purpose was to achieve visualization in a lower dimensional space pointing out similarities and differences among the samples of the original dataset according to their special features. Because PCA is an adaptive technique, the new variables were defined by the dataset at hand, not a priori. Score values in the first three PCs that explained more than 85% of the total variance among reference samples were projected to multiple two-dimensional plots, which were then inspected for possible patterns or groupings. Finally, 8 out of the 20 original intervals, containing in total 264 variables and highest variance in the second and third PCs were selected as model spectral regions for further study.

#### 3.5.3. One-Class Classification

PCA and SIMCA were performed using the SIMCA 14.1 software (Umetrics, Sweden). Data from the selected spectral regions were first scaled according to Pareto method. For the exploratory analysis, only principal components with eigenvalue >1.0 were considered useful, according to the Kaiser criterion [27]. For the one-class classification, the distance to the model (DMoDX) and Hoteling’s *T*^2^ tests (*p* < 0.01) helped to evaluate the class structure and outliers.

### 3.6. GC-Analyses

#### 3.6.1. GC-FID

The GC analyses were accomplished with an Agilent 6890A gas chromatograph equipped with a split-splitless injector and FID (LFCT). Samples were analyzed on a TR-FAME capillary column (60 m × 0.25 mm i.d., film thickness 0.25 μm) (Thermo Scientific, Bellefonte, PA, USA). The carrier gas was helium at a constant flow rate of 2 mL/min. Samples were diluted in dichloromethane 2% (*v*/*v*) and then injected (2 μL) manually onto the GC in split mode with 25:1 ratio. Injector and detector were both kept at 240 °C. The temperature program was 40 °C for 5 min, raised to 100 °C at 15 °C/min, then to 140 °C at 5 °C/min and held for 1 min and finally raised at 240 °C at 15 °C/min and kept for 5 min. Linear regression curve was constructed for 1,8-cineole (y = 187.29x−2130.2, R^2^ = 0.992) within 96–161.21 nmol. Same conditions as described previously were applied. All analyses were performed in triplicate (CV < 5%).

#### 3.6.2. GC-MS

GC-MS analyses were carried out with an Agilent 6890A gas chromatograph equipped with a Mass Selective Detector MSD 5973 mass spectrometer (Agilent Technologies, Palo Alto, CA, USA) and fitted with a DB-WAX capillary column (polyethylene glycol: 30 m × 0.25 mm i.d., 0.33 µm film thickness) (Agilent Technologies, Palo Alto, CA, USA). The transfer line temperature was set at 240 °C. The column carrier gas was helium at a constant flow rate of 2 mL/min. The mass spectrometer was operated in the electron impact mode (EI) at 70 eV, scanning the range 35–350 *m*/*z* at a scan rate of 2.36 scans/s and the ion source temperature was set at 230 °C. Samples (2 μL) were injected manually onto the GC in the split mode at a 25:1 ratio. Solvent delay time was set at 8 min. Gas chromatographic conditions were as reported in the previous paragraph. The volatile constituents were tentatively identified by comparing their elution order and mass spectra with data from the NIST library (Version 2.0f, National Institute of Standards and Technology, Gaithersburg, MD, USA, 2008) and the published literature [35].

## 4. Conclusions

The diagnostic potential of FT-IR fingerprinting of authentic bay laurel essential oils for intra-species variation but also for one-class classification was evidenced in this study. The findings support the introduction of FT-IR as a green analytical technique in the quality control of these precious products, which are often mislabeled and/or adulterated. Stepwise exploratory analysis of the spectroscopic data in parallel with GC-FID-MS compositional analyses were necessary to build a model dataset. This model needs continuous performance evaluation to strengthen its usefulness for future diagnostic applications. Analysis of newly acquired authentic EOs from all producing regions will ensure its validity over time.

## Figures and Tables

**Table 1:** Assignment of the most characteristic bands in the FT-IR transmittance spectra of *L. nobilis* L. EOs based on literature [23,24,25] and spectra of reference compounds.

Wavenumber (cm^−1^)		Assignment	Relevant Constituent(s)
Zero order Spectrum	2nd Derivative Spectrum		
Characteristic group vibrations			
3440	-	*v_s_*(OH)	linalool, terpinene-4-ol, α-terpineol
3073; 2985 (sh)	3075; 2986	*v_s_*(=CH_2_ mono, 1,1) or *v_as_*(CH_2_) in cyclopropyl rings	methyleugenol α-, β-pinene, sabinene, spathulenol, linalool, limonene
2965; 2879	2967; 2879–2870	*v_as_*(CH_3_)	1,8-cineole α-, β-pinene, sabinene, linalool, terpinene-4-ol
	2947–2945	*ν_s_*(CH_3_–C=) or (CH_3_)_2_–C–electronegative or (CH_2_) in cyclobutane	1,8-cineole, other unidentified
2925; 2853 (sh)	2924; 2853	*v_s_*(CH_2_)	sabinene, linalool, β-pinene 1,8-cineole
2834 (sh)	2833	(Ar–CH_2_–O) or Ar–OCH_3_	methyleugenol, eugenol
2724	2725	–CHO	unidentified
1730	1732	*ν*(C=O)	α-terpinyl, bornyl, linalyl acetates
	1713–1695	–C=O–OH or aryl –C(H)=O	alkyl ketones (cyclic), aryl aldehydes
1655–1640 (br)	1660–1630	*v*(C=C) isolated or cyclic	sabinene, linalool, methyleugenol
1514	1516–1514	*v*(C=C) (ring)	methyleugenol, eugenol, *p*-cymene
1440–1510	1467–1465	*v*(C=C–C) (ring) or *δ*(CH_2_)	methyleugenol, eugenol *p*-cymene
Skeletal vibrations			
1446	1445;1433	*δ_s_*(CH_2_) cyclopropyl, cyclobutyl	sabinene, spathulenol, α-, β-pinene
1375–1363	1377; 1364–1360	*v_s_*(CH_3_–C=O) *δs*(CH_3_) gem	1,8-cineole, α-terpinyl acetate
1259; 1167–1155	1262–1258; 1155	*v_as_*(C–O–C) aromatic *v_s_*(C–O–C) aromatic *v*(O=C–O)	methyleugenol, eugenol acetate esters
1080	1080	*v*(C–O–C)	1,8-cineole
1032 (sh)	1033–1031	*v_as_*(CH_2_–O–C=O)	acetates of primary alcohols
1018	1017		α-pinene, γ-terpinene
995	985	*δ*(C–H)	1,8-cineole
	920–916	(CH_3_)_3_–C–O or 5-membered cyclic ethers	
	887	*ω* (C–H) *γ* (=CH_2_)	pinene limonene
	843		
	816	*ω* (C–H)	*p*-cymene
	801–797	*δ*(sp^2^ C–H)	
	770–764	*δ*(sp^2^ C–H)	

*ν*, stretching vibration; *δ*, in plane deformation vibration; *γ*, out of plane deformation vibration *ω*, wagging vibration; sh, shoulder; br, broad.

**Table 2:** GC-MS data for the constituents of bay laurel leaf EOs corresponding to six different sampling dates (M1–M6) from tree D02 (AUTh campus).

No	Compound	Content (%) *					
		D02_M1	D02_M2	D02_M3	D02_M4	D02_M5	D02_M6
	Compounds eluted prior to 8 min not considered						
1	camphene	tr. *	0.51	0.63	0.56	tr.	0.59
2	β-pinene	1.80	2.98	3.35	3.32	2.41	3.97
3	sabinene	4.48	7.23	7.31	8.39	6.36	10.06
4	β-myrcene	0.57	0.76	0.76	1.12	0.99	1.26
5	limonene	1.26	1.72	1.55	1.56	1.80	1.87
6	1,8-cineole	25.6	35.62	24.59	29.92	29.25	34.9
7	γ-terpinene	tr.	0.36	0.37	0.19	tr.	0.39
8	*p*-cymene	tr.	0.49	0.53	0.20	tr.	0.25
9	unidentified	tr.	tr.	0.21	0.25	tr.	0.22
10	unidentified	0.63	0.59	0.48	0.50	tr.	0.68
11	linalool	4.08	3.87	2.48	1.57	1.95	1.34
12	bornyl acetate	1.48	1.13	1.19	0.68	0.98	0.75
13	β-elemene	tr.	0.32	0.68	1.17	1.13	0.39
14	terpinen-4-ol	2.75	2.39	2.38	1.01	2.15	1.75
15	*p*-mentha-1(7),8-diene	1.26	0.98	1.05	0.3	1.07	0.74
16	unidentified	0.61	0.48	0.39	0.38	tr.	0.47
17	terpinyl acetate	31.7	23.62	22.07	15.3	28.39	18.07
18	germacrene D	tr.	tr.	0.50	1.46	1.10	0.39
19	unidentified	0.90	0.60	0.69	tr.	1.04	0.52
20	bicyclogermacrene	0.64	tr.	0.71	4.19	2.73	0.55
21	*δ*-cadinene	tr.	tr.	0.29	1.06	0.97	0.25
22	methyl eugenol	6.42	4.42	4.45	1.80	3.77	2.19
23	ledol	0.66	0.41	0.54	0.4	0.92	0.37
24	unidentified	0.89	0.49	0.78	2.39	1.82	0.63
25	β-guaiene	tr.	1.20	0.60	5.69	1.87	0.68
26	spathulenol	5.25	3.52	4.53	1.90	3.31	2.60
27	eugenol	1.49	1.02	1.25	0.7	1.79	1.52
	Total (%)	92.47	94.71	84.36	86.01	95.8	87.40

* tr.: traces.

## Supplementary Materials

The following are available online, Figure S1: Zero and second (2nd) order derivative FT-IR spectra of EOs obtained from taxonomically identified *Laurus nobilis* leaves, Figure S2: Illustration of i-PCA scoreplots from the pre-processed FT-IR spectra of 97 reference bay laurel EOs. The plots represent sample projections along the first and second, A; first and third, B; second and third, C; principal components and correspond to 4 out of the 8 intervals that were selected for further chemometric analysis: no 1 (599.9–657.7 cm^−1^), no 10 (1138–1195.9 cm^−1^), no 12 (1257.6–1413.8 cm^−1^) and no 20 (3045.6–3101.5 cm^−1^). Taxonomically certified leaf material from •: AUTh campus, •: Athamanon mountain, Epirus, GR •: market. (data for the rest of the intervals are available but not shown), Figure S3: GC-MS chromatographic profiles, of bay laurel leaf EO samples corresponding to six different sampling dates (M1–M6) from tree D02 (AUTh campus). The peaks were cross-referenced against NIST mass spectral library (version 2.0f, 2008) and assigned to compounds no 1–27, as shown in Table 2, Figure S4: Scatterplot of PCA scores (t) along the first and second principal components extracted from the pre-processed FT-IR spectra of 97 reference bay laurel EOs. Taxonomically certified leaf material from •: AUTh campus, •: Athamanon mountain, Epirus, GR •: market. Dot codes refer to the tree (D#) and harvest month (M#) identifiers, Figure S5: GC-MS chromatographic profile of the commercial bay laurel EO coded as VL1. Mass spectra of peaks were cross-referenced against the NIST mass spectral library (version 2.0f, 2008). Identified peaks are shown below, Figure S6: GC-MS chromatographic profiles of some non-laurel commercial EOs examined in this study. Mass spectra of peaks were cross-referenced against the NIST mass spectral library (version 2.0f, 2008). The relative contents of 1,8-cineole were calculated by GC-FID. Designated peak identifiers refer to distinct compounds of the corresponding EOs, Figure S7: FT-IR transmittance spectra of the commercial EO samples (*n* = 21) after 2nd order derivatization and inversion in different sub-regions within 3300–600 cm^−1^. Highlighted regions correspond to spectral bands included in the one-class model for authentic bay laurel EOs, Table S1. GC-MS data for constituents of randomly selected EOs originated from three female and three male bay laurel leaves. The chromatographic peaks were cross-referenced against NIST mass spectral library (version 2.0f, 2008) as explained in 3.6.2, Table S2: Metadata of commercial EOs (*n* = 21), Scheme S1: Work flow diagram for *L. nobilis* one class classification.
