# Supplementary material for: Diagnostic Potential of FT-IR Fingerprinting in Botanical Origin Evaluation of Laurus nobilis L. Essential Oil is Supported by GC-FID-MS Data
Source: Molecules. 2020 Jan 29;25(3):583. doi: 10.3390/molecules25030583 (PMC7037323; doi:10.3390/molecules25030583)
Supplement: Supplementary file 1 [file molecules-25-00583-s001.pdf]

Diagnostic Potential of FT-IR Fingerprinting in Botanical Origin Evaluation of *Laurus nobilis* L. Essential Oil Is Supported by GC-FID-MS Data

Stella A. Ordoudi[1], Maria Papapostolou[1], Stella Kokkini[2] and Maria Z. Tsimidou[1*]

[1] Laboratory of Food Chemistry and Technology (LFCT), School of Chemistry, Aristotle University of Thessaloniki (AUTh), 54124 Thessaloniki, Greece. steord@chem.auth.gr (S.A.O.); papaposm@chem.auth.gr (M.P.); tsimidou@chem.auth.gr (M.Z.T.)

[2] Laboratory of Systematic Botany and Phytogeography (LSBPh), School of Biology, Aristotle University of Thessaloniki (AUTh), 54124 Thessaloniki, Greece. kokkini@bio.auth.gr (S.K)

Supplementary Materials

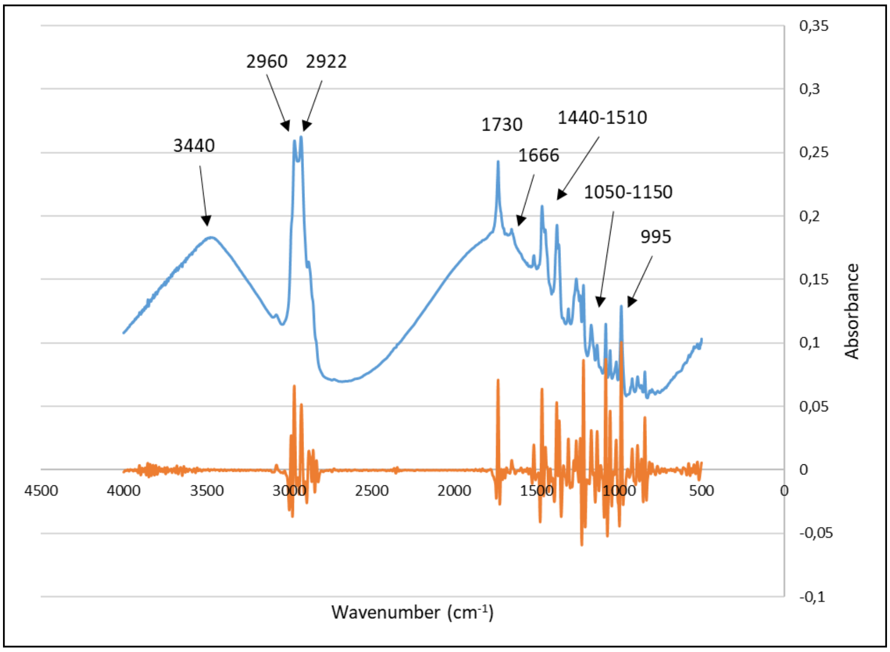

Figure S1. Zero and second (2nd) order derivative FT-IR spectra of EOs obtained from taxonomically identified *Laurus nobilis* leaves.

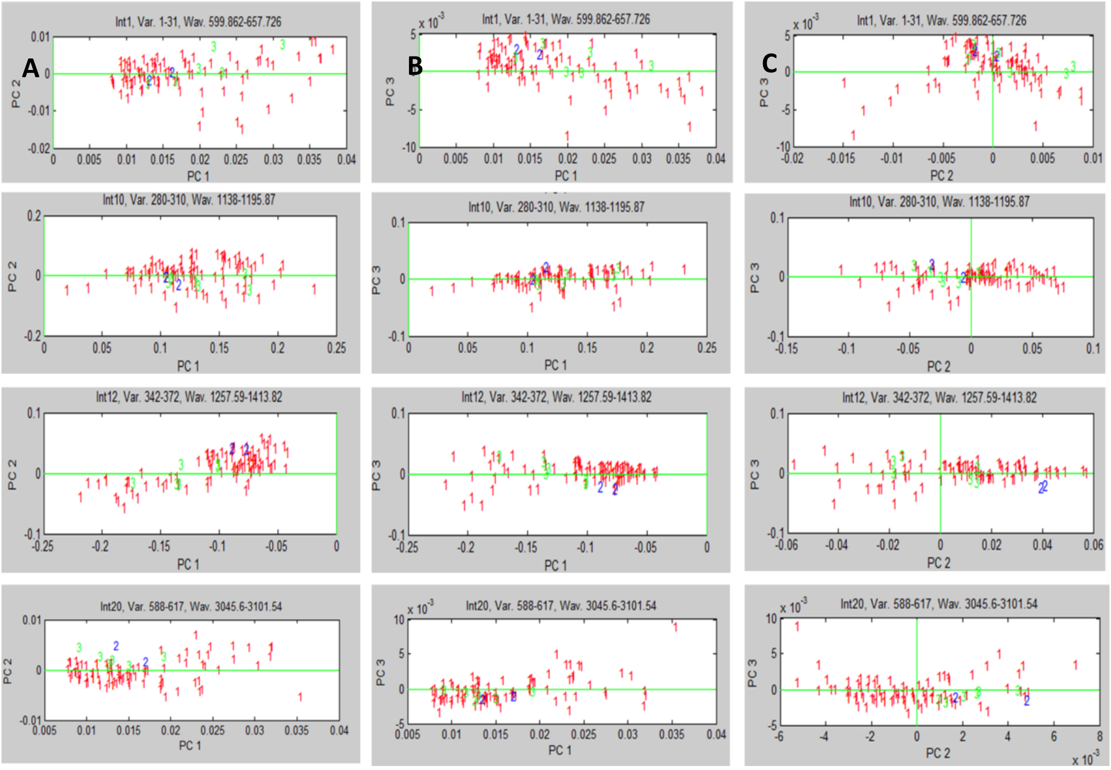

Figure S2. Illustration of i-PCA scoreplots from the pre-processed FT-IR spectra of 97 reference bay laurel EOs. The plots represent sample projections along the first and second, **A;** first and third, **B;** second and third, **C;** principal components and correspond to 4 out of the 8 intervals that were selected for further chemometric analysis: no 1 (599.9–657.7 cm^{-1}), no 10 (1138–1195.9 cm^{-1}), no 12 (1257.6–1413.8 cm^{-1}) and no 20 (3045.6–3101.5 cm^{-1}). Taxonomically certified leaf material from •: AUTh campus, •: Athamanon mountain, Epirus, GR •: market. (data for the rest of the intervals are available but not shown)

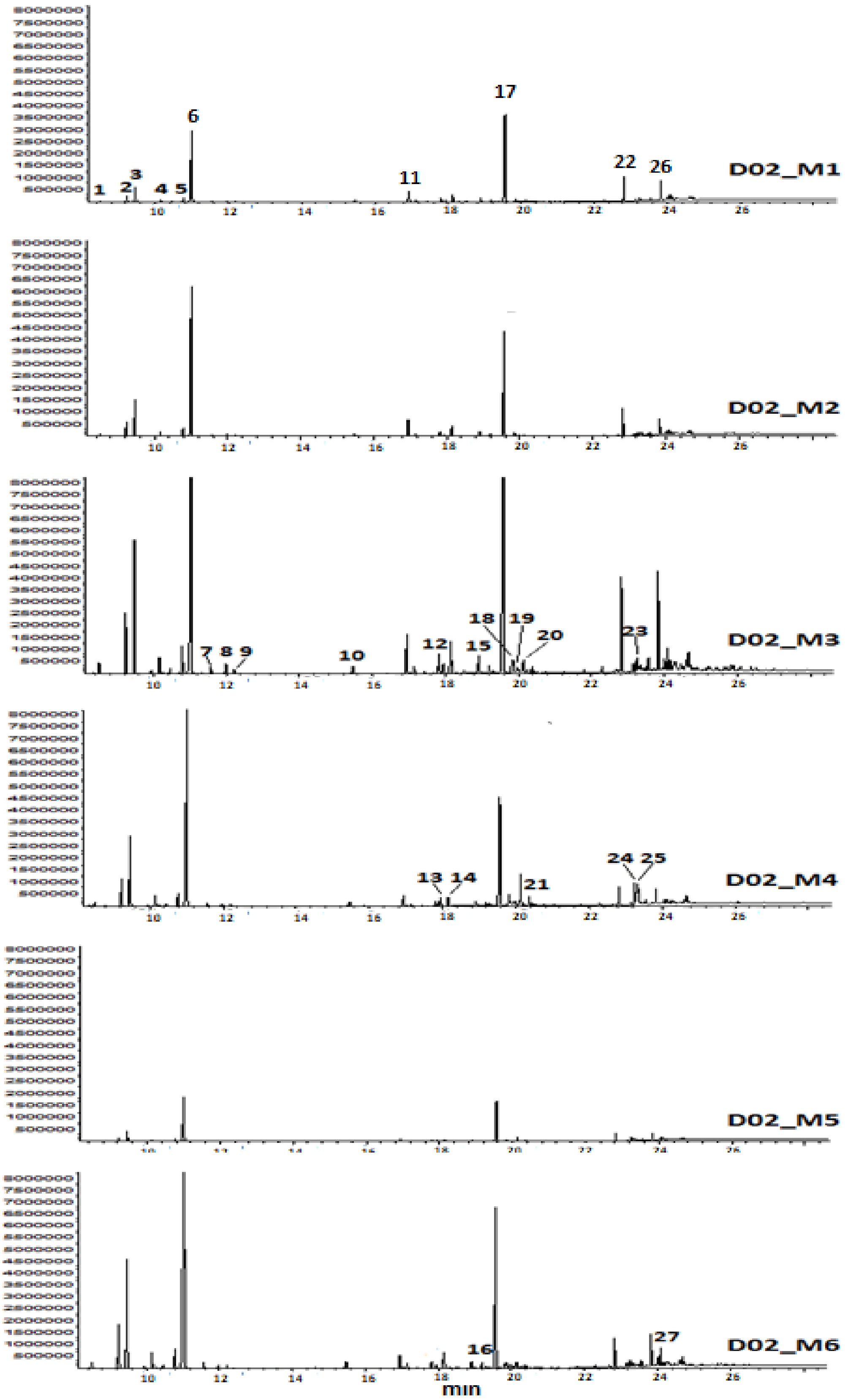

Figure S3. GC-MS chromatographic profiles, of bay laurel leaf EO samples corresponding to six different sampling dates (M1–M6) from tree D02 (AUTh campus). The peaks were cross-referenced against NIST mass spectral library (version 2.0f, 2008) and assigned to compounds no 1–27, as shown in Table 2.

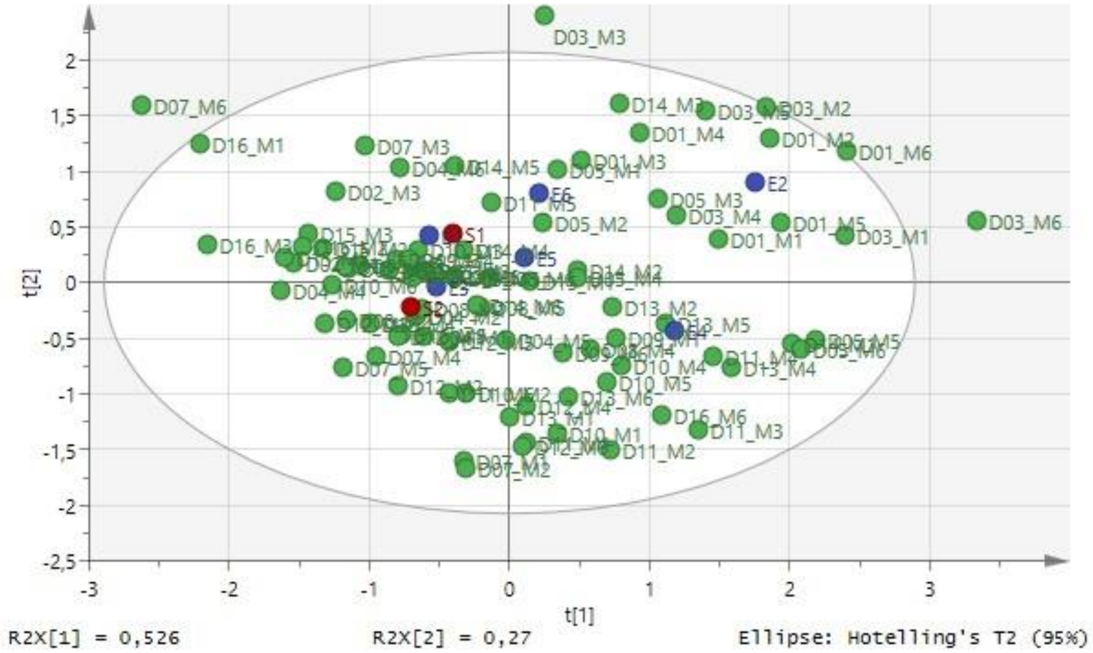

Figure S4. Scatterplot of PCA scores (t) along the first and second principal components extracted from the pre-processed FT-IR spectra of 97 reference bay laurel EOs. Taxonomically certified leaf material from •: AUTh campus, •: Athamanon mountain, Epirus, GR •:market. Dot codes refer to the tree (D#) and harvest month (M#) identifiers.

Table S1. GC-MS data for constituents of randomly selected EOs originated from three female and three male bay laurel leaves. The chromatographic peaks shown in Figure 4, were cross-referenced against NIST mass spectral library (version 2.0f, 2008) as explained in 3.6.2.

No	Compound	Content (%)					
		**D08_M2**	**D04_M3**	**D02_M2**	**D11_M3**	**D03_M2**	**D03_M1**
		**F**	**F**	**F**	**M**	**M**	**M**
	Compounds eluted prior to 8 min not considered						
1	camphene	0.57	0.77	0.51	0.84	1.27	1.09
2	*β*-pinene	3.04	3.98	2.98	3.47	4.51	3.81
3	sabinene	3.20	7.31	7.23	4.52	8.51	7.67
3′**	(+)-3-carene	0.24	tr.	tr.	0.23	0.70	0.60
4	*β*-myrcene	tr.*	0.48	0.76	0.17	0.62	0.57
5	limonene	1.04	1.32	1.72	0.80	1.66	1.48
6	1,8-cineole	40.61	30.11	35.62	34.96	26.6	31.91
7	*γ*-terpinene	0.31	0.42	0.36	0.22	0.32	0.29
8	*p*-cymene	1.78	0.43	0.49	1.20	0.59	0.46
9	unidentified	tr.	0.21	tr.	0.10	tr.	0.17
10	unidentified	0.90	0.56	0.59	0.89	0.53	0.59
11	linalool	1.20	0.76	3.87	1.13	3.83	3.67
11′	pinocarvone	0.40	tr.	tr.	0.63	tr.	tr.
12	bornyl acetate	1.41	1.09	1.13	1.59	2.15	1.77
13	*β*-elemene	0.25	1.05	0.32	0.44	0.31	0.22
14	terpinen-4-ol	3.74	4.39	2.39	3.85	2.06	1.88
14′	unidentified	0.54	0.43	tr.	0.67	0.48	0.40
15	*p*-mentha-1(7),8-diene	1.15	0.69	0.98	0.88	1.26	1.07
16	unidentified	0.58	0.55	0.48	0.64	0.33	0.33
16′	unidentified	0.31	0.52	tr.	0.25	tr.	tr.
17	terpinyl acetate	15.63	19.76	23.62	10.88	23.77	19.12
17′	borneol	tr.	0.31	tr.	tr.	0.31	0.27
18	germacrene D	1.19	0.7	tr.	1.94	tr.	tr.
19	unidentified	tr.	0.64	0.6	tr.	tr.	tr.
19′	unidentified	0.55	0.51	tr.	0.63	0.27	0.25
20	bicyclogermacrene	0.22	0.69	tr.	0.56	0.3	0.28
21	*δ*-cadinene	tr.	0.61	tr.	0.28	0.26	0.30
21′	nerol	0.29	0.31	tr.	0.62	0.29	0.26
22	methyl eugenol	6.35	6.42	4.42	5.36	5.71	5.54
23	ledol	0.22	0.32	0.41	0.36	0.49	0.60
24	unidentified	0.18	0.67	0.49	0.44	0.51	0.52
25	*β*-guaiene	tr.	0.22	1.20	0.25	tr.	tr.
26	spathulenol	0.79	0.59	3.52	1.04	3.06	2.92
27	eugenol	2.93	1.96	1.02	1.77	1.03	1.07
	**Total %**	**89.62**	**81.61**	**88.78**	**91.73**	**94.71**	**89.11**

*traces; **3′, 11′, 14′, 16′, 17′, 19′, 21′ indicate compounds not reported in Figure 3

Table 2. Metadata of commercial EOs (n = 21).

Sample Code	Commercial Name	Origin of Plant Material	Expiry Date	Extra Information on the Label/Producer or Distributor
OL1	Laurel	Greece[2]	Dec 2023	-[4]
OL2	Laurel	Greece[2]	Dec 2023	-[4]
SL1	Laurel	Italy[2]	Feb 2020	-[4]
SL2	Laurel	West Indies[1]	Feb 2020	-[4]
AL1	Daphne	Italy[2]	Dec 2019	-[4]
AL2	Daphne	Unknown[2]	Dec 2020	-[4]
FL1	*Laurus nobilis*	Italy[2]	Apr 2020	-[4]
FL2	*Laurus nobilis*	Italy[2]	Apr 2020	-[4]
EL1	*Laurus nobilis*	Germany[2]	-[4]	-[4]
VL1	Laurel (*Laurus nobilis*)	Greece[2]	-[4]	-[4]
VL2	Laurel (*Laurus nobilis*)	Greece[2]	-[4]	-[4]
OE1	Eucalyptus	Egypt[2]	Dec 2023	-[4]
OE2	Eucalyptus	Egypt[2]	Dec 2023	-[4]
EE	Eucalyptus (*E. globulus*) organic	Portugal[3]	-[4]	*E. globulus* leaf EO, twig oil, limonene Contains 85% cineole, Produced by steam distillation
PE	Eucalyptus oil 80/85 Pharmacopoeia	Unknown[2]	Jan 2021	-[4]
VE	Eucalyptus (*E. globublus*)	Greece[2]	-[4]	-[4]
AE1	Sage	E.U.[1] (Greece[2])	Dec 2020	-[4]
AE2	Sage	E.U.[1]	Dec 2020	-[4]
OR	Rosemary	Egypt[2]	Dec 2023	-[4]
VM1	Melissa (*M. officinalis*)	Greece[2]	-[4]	-[4]
VM2	Melissa (*M. officinalis*)	Greece[2]	-[4]	-[4]

[1]*As indicated on the label;* [2]*Personal communication;* [3]*As mentioned on the website;* [4]*Not available*

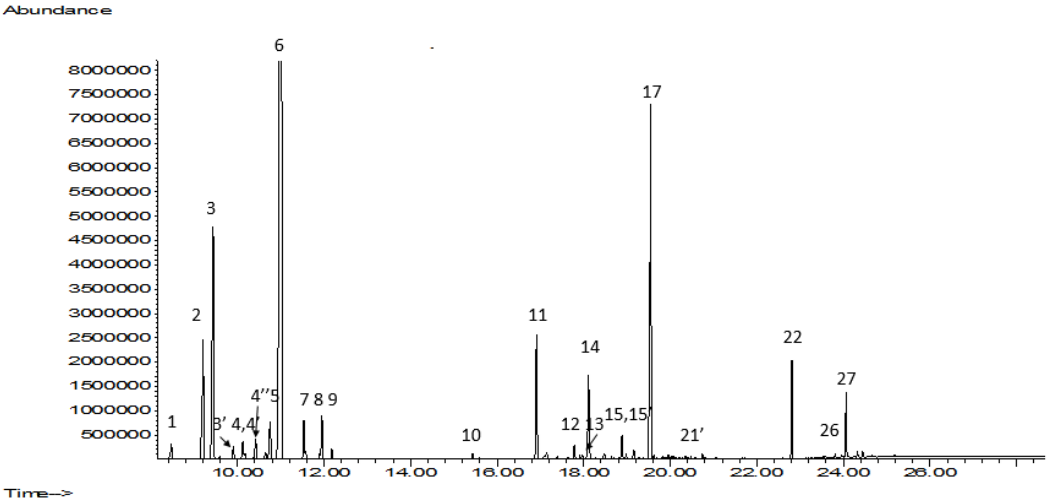

Figure S5. GC-MS chromatographic profile of the commercial bay laurel EO coded as VL1. Mass spectra of peaks were cross-referenced against the NIST mass spectral library (version 2.0f, 2008). Identified peaks are shown in the insert below.

No	Compound	Content (%) VL1
	Compounds eluted prior to 8 min not considered	
1	camphene	0.48
2	*β*-pinene	3.77
3	sabinene	6.51
3′	(+)-3-carene	3.42
4	*β*-myrcene	0.53
4′	*α*-phellandrene	0.25
4′′	(+)-2-carene	0.45
5	limonene	3.68
6	1,8-cineole	45.96
7	*γ*-terpinene	1.14
8	*p*-cymene	3.89
9	unidentified	0.4
10	unidentified	0.19
11	linalool	3.35
12	bornyl acetate	0.52
13	*β*-elemene	0.09
14	terpinen-4-ol	2.32
15	*p*-mentha-1(7),8-diene	0.24
15′	estragole	0.44
17	terpinyl acetate	14.27
21′	nerol	0.44
22	methyl eugenol	4.54
26	spathulenol	0.08
27	eugenol	1.36
	**Total %**	98.32

*3′, 4′, 4′′, 15′, 21′ indicate compounds not reported in Figure 3

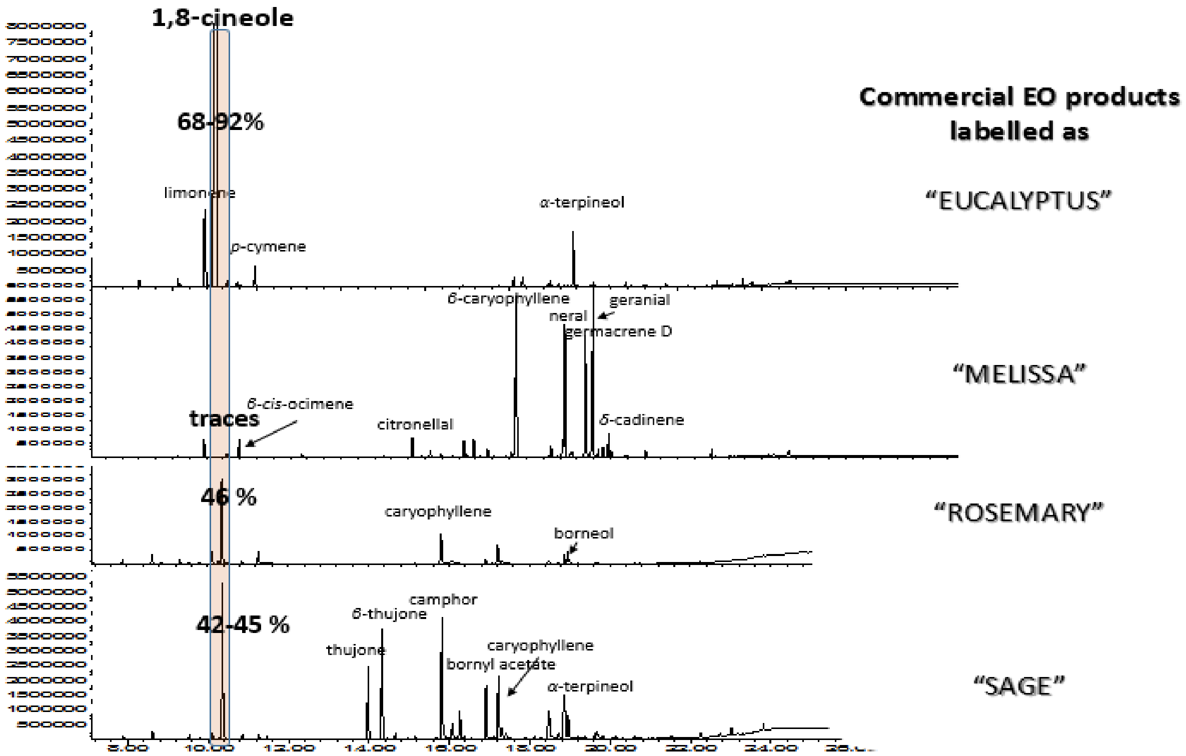

Figure S6. GC-MS chromatographic profiles of some non-laurel commercial EOs examined in this study. Mass spectra of peaks were cross-referenced against the NIST mass spectral library (version 2.0f, 2008) The relative contents of 1,8-cineole were calculated by GC-FID. Designated peak identifiers refer to distinct compounds of the corresponding EOs

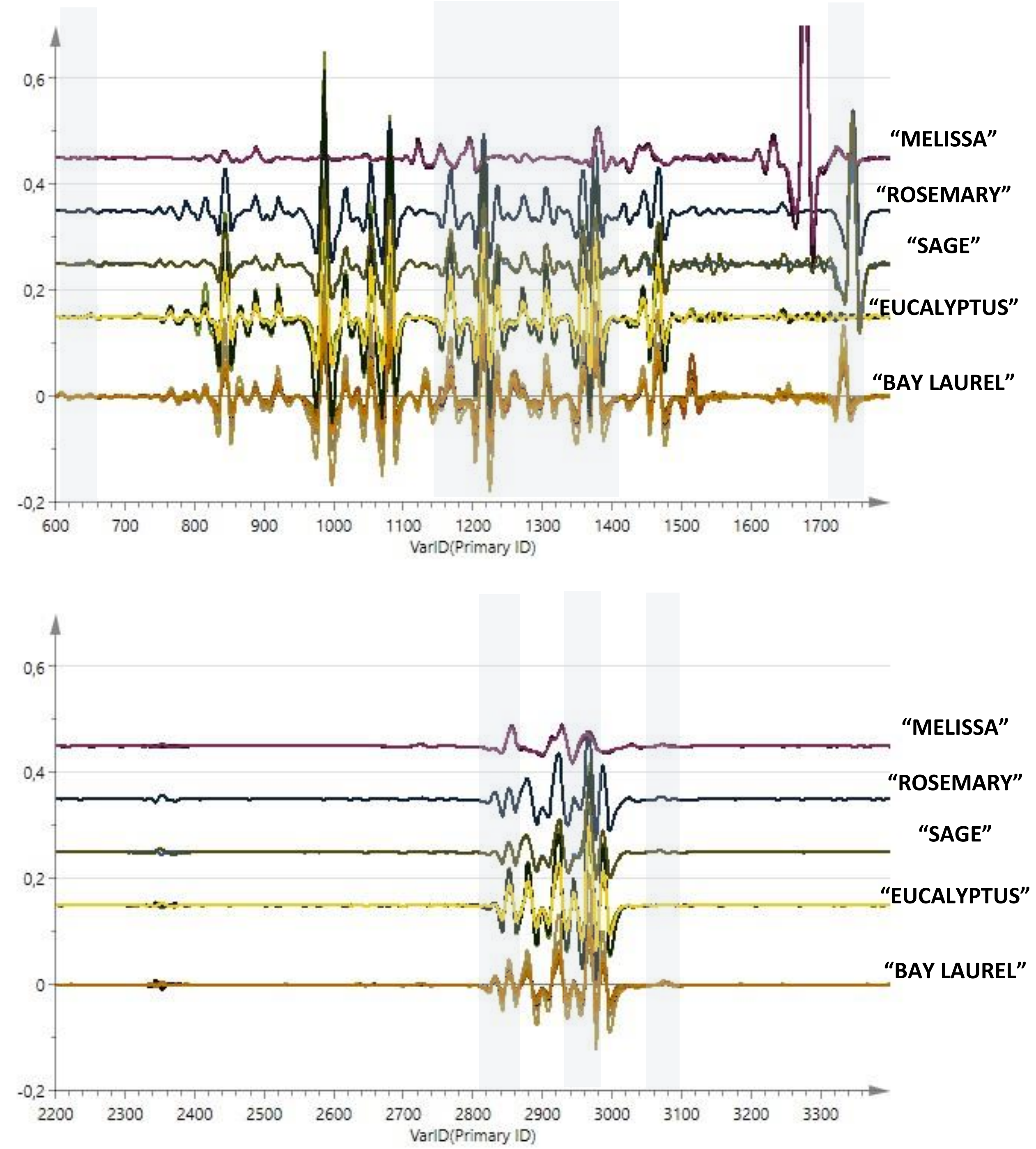

Figure S7.FT-IR transmittance spectra of the commercial EO samples (*n* = 21) after 2nd order derivatization and inversion in different sub-regions within 3300 - 600 cm^{-1}. Highlighted regions correspond to spectral bands included in the one-class model for authentic bay laurel EOs.

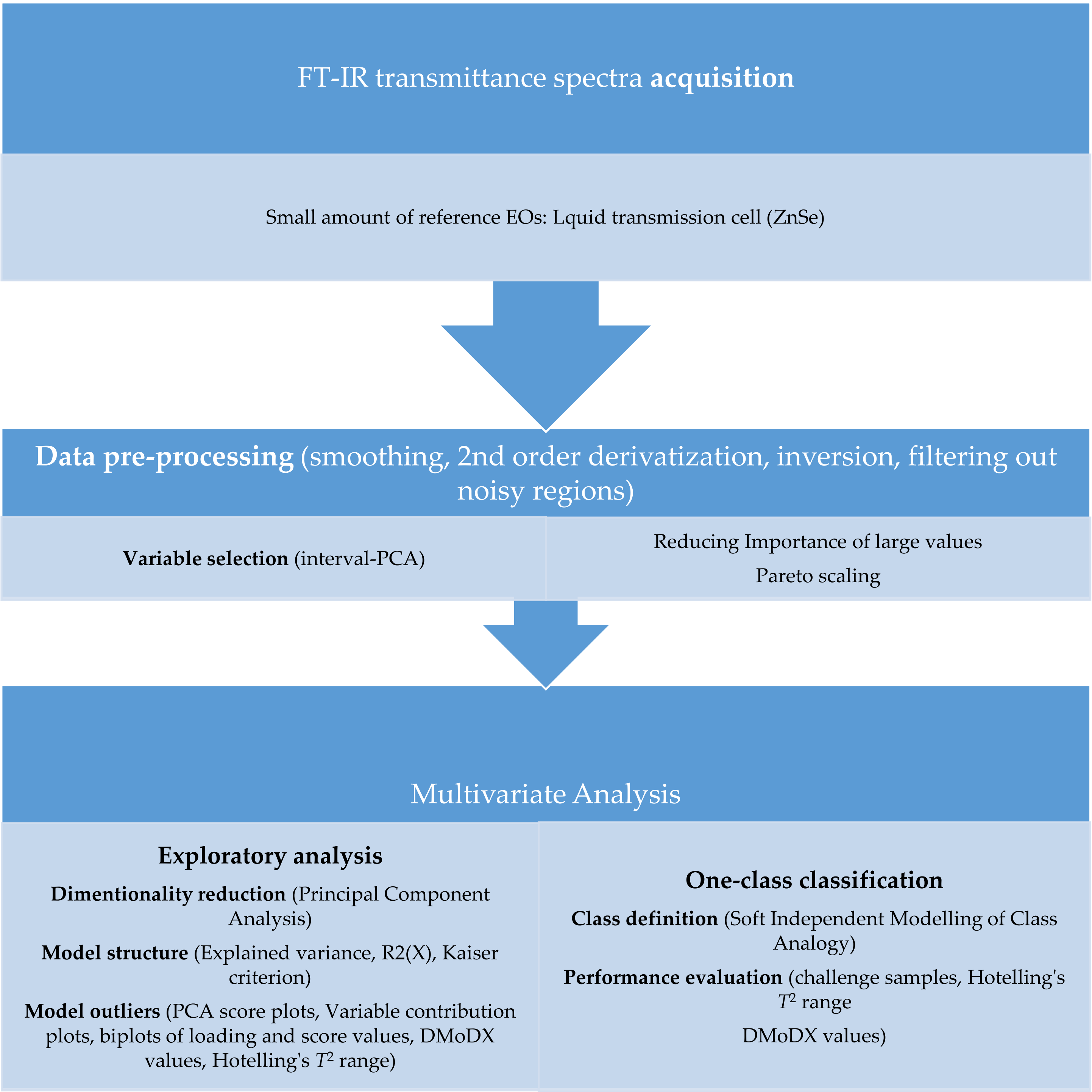

Scheme S1. Work flow diagram for *L. nobilis* one class classification
